# FLT3 Testing and Guideline Concordance in Acute Myeloid Leukemia Across a Community Health System

**DOI:** 10.7759/cureus.108034

**Published:** 2026-04-30

**Authors:** Carley Yawn, Logan Van Ravenswaay, Sarah Nisly, David Reeves

**Affiliations:** 1 Insights, Decera Clinical, Reston, USA; 2 College of Pharmacy, Butler University, Indianapolis, USA

**Keywords:** acute myeloid leukemia, flt3 mutation, guideline concordance, real-world evidence, targeted therapy

## Abstract

Introduction: FMS-like tyrosine kinase 3 (FLT3) mutations are present in approximately one-third of patients with acute myeloid leukemia (AML) and are associated with higher relapse and reduced survival. National Comprehensive Cancer Network (NCCN) guidelines recommend FLT3 mutation testing at diagnosis to guide risk stratification and selection of FLT3-targeted therapy. However, real-world adherence to these recommendations remains variable. The objective of this mixed-methods study was to evaluate the rates of FLT3 testing and guideline-concordant treatment within a community health system, and to identify the clinical and systemic factors driving treatment decision-making.

Methods: A retrospective, mixed-methods cohort study was conducted using anonymized data from adult patients diagnosed and treated for AML between 2017 and 2024 within a multi-site community health system. The quantitative component included FLT3 testing status, mutation subtype, induction regimen, FLT3 inhibitor use, and therapy outcomes. Guideline concordance was assessed based on relevant NCCN recommendations and FDA-approved treatments at the time of diagnosis. Quantitative analyses, including descriptive statistics and time-to-event analyses, were performed using IBM SPSS Statistics for Windows, version 29 (IBM Corp., Armonk, NY, USA). The qualitative component consisted of clinician focus groups conducted after quantitative data collection and analysis to examine real-world molecular testing workflows, treatment decision-making, and barriers to guideline-concordant care. Focus group transcripts were analyzed using a thematic approach, and qualitative findings were integrated with quantitative results to contextualize observed practice patterns.

Results: A total of 210 AML patients were included. Overall, 78.1% (n = 164) of induction regimens for all patients with AML were guideline concordant based on year of diagnosis/treatment. Deviations from guideline concordant care most frequently occurred when treatment was initiated prior to the availability of FLT3 results or when emerging regimens were used before formal guideline inclusion. FLT3 testing at diagnosis was documented in 83.8% (n = 176) of patients. Among those patients tested, 23.9% (n=42) were FLT3-positive, with internal tandem duplication (ITD) mutations being the most common.

Qualitative findings were derived from two clinician focus group sessions. Thematic analysis of clinician focus groups identified three key themes highlighting variability in clinical decision-making by specialty, inconsistencies in molecular testing workflows and system-level constraints, and evolving preferences toward newer FLT3 inhibitors.

Conclusion: From 2017 to 2024, FLT3 testing and guideline-concordant treatment were commonly implemented within this community health system, as demonstrated by real-world data derived from the electronic medical record (EMR). The findings highlight strengths in baseline molecular testing and treatment selection, while also identifying opportunities to improve, including in community-based settings. Opportunities remain to improve documentation practices, transitions of care, and molecular retesting at relapse. These findings support targeted quality improvement efforts to optimize molecular testing workflows and treatment alignment in AML.

## Introduction

Acute myeloid leukemia (AML) is a biologically heterogeneous hematologic malignancy that continues to be associated with poor outcomes despite substantial advances in diagnostic and therapeutic strategies over the past decades [1]. In 2025, there were an estimated 22,100 new cases of AML in the United States, with an anticipated five-year relative survival rate of 32.9% [2]. Mutations in the gene encoding FMS-like tyrosine kinase 3 (FLT3) represent one of the most clinically relevant molecular abnormalities in AML. FLT3 internal tandem duplication (FLT3-ITD) mutations, first described in the late 1990s, occur in approximately one-quarter of newly diagnosed AML cases, while FLT3 tyrosine kinase domain (FLT3-TKD) point mutations are present in a smaller subset of patients [3-6]. These mutations result in FLT3 activation, driving downstream signaling pathways that promote leukemic proliferation, inhibit differentiation, and impair apoptosis, ultimately contributing to higher relapse rates and inferior overall survival compared to patients without the mutation [5,7,8].

Current guidelines recommend FLT3 testing be conducted in all untreated patients at the initial time of diagnosis, regardless of age. FLT3 retesting is also recommended for patients with refractory AML [9]. FLT3 inhibitors are standard of care for newly diagnosed FLT3-mutated AML and are recommended by the National Comprehensive Cancer Network (NCCN) Guidelines as part of frontline therapy [10-12]. Midostaurin was the first FLT3 inhibitor to demonstrate improved outcomes when combined with standard induction and consolidation chemotherapy, leading to its approval in 2017 and incorporation into frontline treatment of FLT3-mutated AML [1]. Subsequently, second-generation FLT3 inhibitors, including gilteritinib and quizartinib, have expanded therapeutic options in both relapsed/refractory and newly diagnosed settings [1]. Gilteritinib is currently indicated for relapsed/refractory disease, and quizartinib is indicated only for ITD mutations [13,14]. For those with an FLT3-ITD mutation, treatment with midostaurin or quizartinib in combination with chemotherapy is generally recommended [12]. Although these agents have not been directly compared, a recent retrospective multicenter cohort study of five academic hospital systems found that for patients with an FLT3-ITD mutation, those treated with quizartinib were more likely to achieve composite complete remission, less likely to have dosing interruptions, and less likely to require reinduction compared to those receiving midostaurin [15].

Despite these advances, disease relapse remains a significant challenge, driven in part by clonal evolution and resistance mechanisms that allow leukemic cells to escape FLT3 inhibition [8]. Recently published literature has revealed that not all patients are being tested for FLT3 mutations upon initial diagnoses and after relapses or recurrences. In a study conducted by Gong et al., only 63% of patients were screened for FLT3 mutation, and initial treatment decisions appeared unaffected by the patient’s FLT3 status. In this study, testing FLT3 status in newly diagnosed AML was shown to increase from 2016 to 2019, but the rate for retesting among relapsed/refractory (R/R) AML remained a persistent gap [16]. Furthermore, few studies have evaluated the differences in FLT3 screening between academic health centers and community practice centers, with Lin et al. reporting that molecular testing was more frequently completed in academic centers versus community practice centers [17].

As a result, optimal integration of FLT3 inhibitors-particularly balancing established agents such as midostaurin with newer, more selective therapies-remains an area of active clinical decision-making. Therefore, the primary objective of this mixed-methods study was to quantify real-world FLT3 testing rates and guideline-concordant therapy utilization within a multi-site community health system. The secondary objective was to qualitatively explore the clinical workflows, system-level barriers, and provider preferences influencing these practice patterns.

## Materials and methods

Study design and setting

This retrospective, mixed-methods cohort study evaluated adult patients (≥18 years of age) diagnosed with AML and treated with AML-directed therapies between January 1, 2017, and December 31, 2024, across a multi-site community health system. The health system comprised 12 hospital campuses, offering comprehensive cancer services to central, northwest, and western Indiana, plus south suburban Chicago in Illinois. Data were extracted from the Epic electronic medical record (EMR) system with the Beacon oncology module (Epic Systems Corporation, Verona, WI) utilizing SlicerDicer (Epic Systems Corporation). All patient data were de-identified prior to analysis. The study period was selected to capture changes in treatment patterns following the approval of midostaurin in 2017 and subsequent emergence of newer FLT3-targeted therapies.

Cohort identification and search strategy

Patients were identified using a structured EMR query that included myeloid leukemia ICD 10: C92 myeloid Leukemia and received drugs utilized in the treatment of AML (daunorubicin, idarubicin, cytarabine, daunorubicin/cytarabine liposomal, midostaurin, quizartinib, fludarabine, gemtuzumab ozogamicin, azacitidine, decitabine, venetoclax, gilteritinib) [18].

Eligibility criteria are summarized in Table 1.

**Table 1:** Inclusion and exclusion criteria

Inclusion criteria	Exclusion criteria
Age ≥ 18 years at the time of AML diagnosis	Received induction therapy at an outside facility (e.g., transferred at relapse or for transplant)
Diagnosis of acute myeloid leukemia (AML)	Initiated induction therapy outside the predefined study period
Order for AML-directed therapy within the health system	Did not ultimately receive AML-directed therapy despite AML diagnosis
Initiation of therapy between January 1, 2017, and December 31, 2024	—

Data collection

Collected variables included age at diagnosis, sex, FLT3 testing status at diagnosis, FLT3 mutation subtype, induction regimen, use of FLT3 inhibitors, relapse occurrence, repeat FLT3 testing at relapse, and survival status. Guideline concordance was assessed by comparing induction regimens with NCCN recommendations and approved treatments at the time of diagnosis [11].

Quantitative analysis

Descriptive statistics were used to summarize patient characteristics and induction treatment patterns. Categorical variables were reported as frequencies and percentages, and continuous variables as medians with ranges. Kaplan-Meier methods were used to evaluate time to relapse in all 210 patients diagnosed with AML between 2017 and 2024. Time-to-relapse was defined as the interval from the date of initial AML diagnosis to the date of documented relapse. All patients who were assessed to have a relapse were included, and all patients who did not experience relapse during the study period were censored in the analysis. These findings directly informed the development of multidisciplinary focus group discussions, in which longitudinal EMR data were presented to healthcare professionals (HCPs) to guide structured dialogue around barriers, practice variation, and workflow inefficiencies. Quantitative analyses, including descriptive statistics and time-to-event analyses, were performed using IBM SPSS Statistics for Windows, version 29 (IBM Corp., Armonk, NY, USA).

Qualitative analysis

Clinician focus groups were conducted after preliminary quantitative analyses to explore molecular testing workflows, treatment decision-making, and barriers to guideline-concordant care. The qualitative component consisted of two focus group sessions involving a total of seven HCPs. Participants were recruited based on their clinical involvement in the management of patients with AML; selection was informed by relevant clinical expertise and health-system location.

A semi-structured discussion guide was used for both focus groups; the full guide is provided in Appendix A. Core domains included: (1) workflows for ordering and documenting molecular testing; (2) how FLT3 results informed induction and relapse treatment decisions; (3) barriers to timely testing or guideline-concordant treatment; (4) differences in decision-making across provider roles or sites; and (5) perceptions of newer FLT3 inhibitors and areas needing additional guidance.

AI-assisted thematic analysis and human oversight

Focus group discussions were audio recorded, transcribed verbatim, and analyzed using a thematic analysis approach. Qualitative coding and synthesis were supported using a large language model (LLM)-assisted analytic workflow (ChatGPT, OpenAI, San Francisco, CA, USA; paid version 5.2) to aid in initial theme identification and narrative synthesis. The final interpretation remained investigator-led.

The standardized prompts used in the analytic workflow were as follows: Prompt 1: “Review the following focus group transcript and generate an initial list of recurring concepts related to FLT3 testing workflows, treatment decision-making, and barriers to guideline-concordant AML care. Do not infer beyond the transcript.” Prompt 2: “Group the identified concepts into three main themes. For each theme, provide representative supporting quotations and note any contradictory views.” Prompt 3: “Draft a concise narrative summary of the major themes, preserving participant meaning and distinguishing workflow barriers, treatment-selection factors, and perceptions of newer FLT3 inhibitors.”

Human oversight was incorporated at multiple stages. One reviewer (CY) conducted a primary review of transcripts and thematic assignment. Identified themes were iteratively reviewed by two other authors (DR, SN) to ensure alignment with the source transcripts and quantitative findings. Formal blinded dual coding was not performed; instead, discrepancies in thematic interpretation were resolved through the iterative review and refinement of analytic prompts and outputs. Final qualitative findings were triangulated with quantitative analyses to contextualize observed patterns of care and implementation challenges.

## Results

Quantitative results

Study Population and FLT3 Testing

A total of 340 adult patients met the inclusion criteria for screening. Of these, 94 were excluded because they received induction at an outside facility (transferred in at relapse or for transplant), 31 initiated induction therapy outside of the predefined study period, and five did not ultimately receive AML-directed therapy despite an AML diagnosis.

After application of these exclusion criteria, the final study cohort included 210 adult patients with newly diagnosed AML and a median age at diagnosis of 66 (IQR: 56-73) years. Most patients underwent documented FLT3 testing at diagnosis, though incomplete documentation was noted in a subset of cases (Table 2).

**Table 2:** FLT3 testing status (N = 210) FLT3: FMS-like tyrosine kinase 3, ITD: internal tandem duplication, TKD: tyrosine kinase domain

FLT3 status	% (n)
Positive​ - ITD	16.7 (35)
Positive - TKD	3.3 (7)
Negative​	62.9​ (132)
Not tested​	1.4​ (3)
​Undocumented	15.7 (33)

Induction Therapy and Guideline Concordance

The most frequently used induction therapies included venetoclax in combination with a hypomethylating agent (30%, n = 63), 7+3 (15.7%, n = 33), and a combination regimen consisting of fludarabine, cytarabine, idarubicin, and granulocyte colony-stimulating factor (FLAG IDA; 13.8%, n = 29). FLT3 inhibitors were administered during induction to 85.7% (n = 36) of patients with FLT3 mutations (n = 42). The most commonly used regimen in the study period for patients with FLT3 mutations was 7+3 in combination with midostaurin (40%, n = 17). Key trends in induction treatment patterns by year and age are shown in Table 3. A full analysis of treatment patterns year-over-year is shown in Appendix B.

**Table 3:** Induction treatment patterns by year and age (N = 210) FLAG-IDA: fludarabine, cytarabine, idarubicin, granulocyte colony-stimulating factor, HMA: hypomethylating agent, FLT3: FMS-like tyrosine kinase 3

Category		Dominant regimens	Key trend
Treatment patterns by year	2017–2018	7+3; 7+3 + midostaurin	Initial midostaurin adoption
	2019–2020	7+3 ± midostaurin; FLAG-IDA	Increased molecular-guided therapy
	2021–2022	Venetoclax + HMA; 7+3 + midostaurin	Expansion of lower-intensity regimens
	2023–2024	Venetoclax-based regimens; quizartinib	Early adoption of newer FLT3 inhibitors
Treatment patterns by age	Age <60 years	7+3 ± midostaurin; FLAG-IDA	High-intensity favored
	Age 60–69 years	7+3 ± midostaurin; venetoclax + HMA	Mixed intensity
	Age ≥70 years	Venetoclax + HMA; HMA alone	Lower-intensity favored

Overall, 78.1% (n = 164) of all patients diagnosed with AML received guideline-concordant therapy. Guideline concordance for patients with FLT3 mutations was 80.9% (n = 170). The most common reasons for guideline discordance were initiating treatment without known FLT3 mutation status and the addition of venetoclax to intensive induction regimens prior to its inclusion in the NCCN guidelines as a Category 1 recommendation in the 2021 update [9]. Within all patients with AML (N=210), disease relapses (Table 4) occurred in patients with a median time to relapse of 288 (IQR: 139-502) days.

**Table 4:** Clinical outcomes, relapse, and FLT3 retesting among patients with AML (N = 210) †Outcome categories were not mutually exclusive, and patients could contribute to more than one category. *Initial treatment success is defined as the achievement of remission following the first intensive induction therapy without the need for an immediate second intensive induction cycle or, if receiving lower intensity therapy, response to the initial lower intensity regimen. FLT3: FMS-like tyrosine kinase 3, AML: acute myeloid leukemia

Outcome^†^	% (n)
Initial treatment success*	61.4 (129/210)
Disease relapse	27.1 (57/210)
Repeat FLT3 testing	77.2 (44/57)
Alive at end of data collection (April 1, 2025)	26.2 (55/210)
Lost to follow-up/transferred care	20.0 (42/210)

Qualitative results

Preliminary thematic analysis yielded six categories, which were consolidated into three, higher-order, actionable themes to capture patterns and reduce redundancy across clinician perspectives. This consolidation process emphasized areas where multiple themes converged on the same underlying barriers or decision points, particularly those with direct implications related to FLT3 testing, treatment selection, and system-level factors influencing care delivery (Table 5).

**Table 5:** Integrated qualitative themes from clinician focus groups FLT3: FMS-like tyrosine kinase 3, EMR: electronic medical record, NGS: next-generation sequencing, MRD: minimum residual disease, TKD: tyrosine kinase domain, ITD: internal tandem duplication

Theme	Description	Focus group participant findings
Fragmentation of clinical decision-making	Treatment selection varied by provider specialty and role, with oncology teams favoring established agents and transplant teams favoring newer FLT3 inhibitors.	“Oncology still defaults to midostaurin, but transplant almost always prefers quizartinib.”
Molecular testing workflows and system-level constraints	FLT3 testing was routinely ordered, but responsibility for ordering, assay selection, and documentation varied. Limited access to rapid or high-sensitivity assays, reliance on send-out testing, and EMR-related gaps affected timing and downstream decisions.	“It’s not always clear who’s responsible for ordering which tests.”; “Most of this is send-out—if it’s Friday, things can get delayed.”; “We’re relying on NGS for MRD because we don’t have access to flow-based MRD.”
Evolving FLT3 inhibitor preferences	Increasing comfort with quizartinib and gilteritinib, with uncertainty around TKD mutations and lower-intensity regimens.	“Quizartinib has become our go-to for ITD.”

Decisions regarding induction regimen, FLT3 inhibitor selection, and sequencing were frequently individualized, shaped by provider experience, specialty norms, and referral context rather than shared standards. This was especially evident at transitions of care, such as from oncology to transplant, where patients often arrived having received heterogeneous regimens, requiring downstream teams to adapt rather than build on a consistent treatment plan. HCPs described this variability as inefficient and potentially confusing, emphasizing that it stemmed less from disagreement with evidence and more from the absence of a unified, institution-wide framework to guide FLT3-directed care.

While FLT3 mutation testing itself was routinely performed, HCPs highlighted persistent uncertainty around responsibility for ordering, assay selection, and interpretation, particularly when multiple platforms or send-out laboratories were involved. These process gaps were compounded by limited access to high-sensitivity measurable residual disease (MRD) assays, leading HCPs to rely on next-generation sequencing (NGS)-based variant allele frequency despite recognizing its limitations. As a result, MRD findings were often viewed as informative but not actionable, restricting their use in decisions about maintenance duration, transplant timing, or therapy discontinuation. EMR-related inconsistencies further obscured molecular data, creating challenges for both real-time decision-making and retrospective quality assessment.

Across HCPs, there was clear momentum toward newer FLT3 inhibitors, particularly quizartinib, driven by emerging data, transplant feasibility, and perceived tolerability advantages. However, this shift was uneven and accompanied by unresolved questions, especially regarding TKD-only mutations and integration of FLT3 inhibitors with lower-intensity regimens such as azacitidine/venetoclax. HCPs expressed discomfort extrapolating evidence into these scenarios, leading to variable practices and frequent reliance on ad hoc tumor board discussions. This uncertainty reinforced calls for targeted education and consensus guidance to support confident adoption of newer agents without increasing practice variability.

## Discussion

In this real-world, mixed-methods analysis of patients with AML treated across a multi-site community health system over a seven-year period, we observed high rates of FLT3 molecular testing and substantial adherence to guideline-recommended induction therapy. These findings support other research in this space that comprehensive molecular diagnostics and targeted treatment strategies based on guideline-based care are being implemented not only in academic centers, but also within community-based oncology practices, with high guideline concordance.

Differences between the present findings and prior real-world reports are likely driven in part by methodological factors. Earlier studies evaluating FLT3 testing practices were conducted during periods when molecular testing and FLT3-directed therapies were not yet routinely integrated into AML care and often relied on registry-based data with limited clinical granularity. In contrast, this cohort included more than two hundred patients diagnosed between 2017 and 2024, a period marked by rapid evolution in AML treatment. Additionally, this cohort utilized detailed EMR review within a single, integrated community health system, enabling assessment of both molecular testing practices and downstream treatment selection. Additionally, whereas prior reports primarily focused on diagnostic testing rates or academic-community comparisons, the present study evaluated guideline concordance across the full treatment continuum, including induction therapy and FLT3 inhibitor use.

Baseline characteristics reflected an older population typical of AML, with diverse induction approaches reflecting patient fitness, disease biology, and evolving standards of care. Importantly, documented FLT3 testing was performed in the majority of patients at diagnosis. In cases where FLT3 results were not explicitly recorded in the EMR, other molecular testing was often documented, suggesting that testing was performed but incompletely captured. This highlights documentation and reporting gaps rather than the true absence of molecular evaluation.

Overall guideline concordance was high, including appropriate use of FLT3 inhibitors in patients with identified FLT3 mutations. When guideline discordance occurred, it most commonly reflected initiation of therapy prior to availability of FLT3 results or use of regimens supported by emerging evidence that had not yet been incorporated into NCCN guidelines at the time of treatment [12].

Patterns of FLT3 inhibitor use observed in this study must be interpreted in the context of drug availability and approval timelines. Midostaurin, approved in 2017, was available throughout the entire study period and consequently represented the most frequently utilized FLT3 inhibitor. Gilteritinib, approved in 2018, became available early in the study period, while quizartinib received United States Food and Drug Administration approval in late 2023, limiting its representation within this dataset. Qualitative findings from clinician focus groups further contextualized these patterns, revealing a degree of clinical inertia favoring established agents, particularly among general oncology providers. Conversely, transplant specialists reported greater familiarity and comfort with newer FLT3 inhibitors, especially in the post-transplant maintenance setting, though these observations reflect perspectives from a small, single-system sample and may not represent broader practice patterns.

Mixed-methods integration of the focus group data provided important insight into decision-making processes that were not apparent from quantitative data alone. HCPs consistently emphasized the importance of molecular testing at diagnosis, described institutional workflows that support timely testing even in community settings, and acknowledged variability in treatment selection based on subspecialty, experience, and perceived toxicity profiles. Advanced practice providers frequently described a “go-with-the-flow” approach, in which treatment decisions were guided by attending physician preference or established service norms, whereas physicians more often articulated active deliberation regarding regimen selection and incorporation of newer agents.

Limitations

Several important limitations should be considered when interpreting these findings. First, this was a retrospective observational study conducted within a single integrated community health system, which may limit generalizability to other practice environments, including independent community practices or large academic centers. Although the single system, multi-site design improves representativeness, institutional workflows, formulary access, and provider expertise may differ elsewhere.

Second, the study period spanned rapid therapeutic evolution in AML. Guideline recommendations, drug approvals, and emerging evidence changed substantially between 2017 and 2024. Although concordance was assessed based on recommendations at the time of diagnosis, retrospective interpretation may not fully capture real-time clinical decision complexity.

Third, incomplete or undocumented FLT3 mutation data represent a meaningful limitation. While 83.8% of patients had documented FLT3 testing results, 15.7% had an undocumented FLT3 status, and 1.4% were not tested according to the EMR. Estimates of FLT3 testing rates and guideline concordance may be overestimated if patients with undocumented mutation status differed systematically from those with clearly documented results. Because undocumented cases could not be reliably reclassified retrospectively, no sensitivity analyses were performed, and findings related to testing rates and concordance should therefore be interpreted with caution.

Fourth, internal validity of the study may have been impacted by selection bias and residual confounding. Patients who initiated induction therapy at an outside facility-most commonly those transferred for relapse management or transplant evaluation-were excluded, accounting for approximately one‑quarter of initially screened patients. This exclusion likely resulted in a cohort enriched for patients receiving initial induction within the health system and may therefore overrepresent more standardized or less complex induction scenarios, potentially inflating observed rates of guideline concordance and limiting applicability to referral‑heavy or tertiary‑care populations. In addition, the quantitative analyses were primarily descriptive and did not adjust for key clinical or system‑level confounders such as comorbidity burden, disease risk stratification, fitness for intensive therapy, or provider‑level factors. As a result, causal inferences regarding the relationships between FLT3 testing practices, treatment selection, and clinical outcomes cannot be made.

Finally, the qualitative component included a small number of participants (n=7) from within the same health system. While thematic saturation was achieved within this context, perspectives may not reflect broader national practice patterns. While transparency around prompts and investigator oversight was provided, outputs generated by large language models (LLMs) are inherently nondeterministic. Although formal blinded dual coding was not performed, transcripts were analyzed using a structured prompt-based workflow in which the LLM was sequentially guided to (1) identify recurring concepts, (2) group concepts into overarching themes, and (3) generate narrative summaries, after which outputs were reviewed and refined by investigators through consensus. These factors may limit reproducibility and introduce analytic bias, underscoring the exploratory and hypothesis-generating nature of the qualitative findings.

Despite these limitations, the study’s findings underscore the success of molecular testing implementation across diverse practice settings while also identifying opportunities for quality improvement. Standardization of documentation, improved communication of molecular results, and targeted education around newer FLT3 inhibitors may further enhance guideline concordance and optimize patient outcomes. As additional targeted therapies enter clinical practice, continued evaluation of real-world adoption and alignment with evidence-based recommendations will remain essential.

## Conclusions

From the study period, 2017 to 2024, FLT3 testing and guideline-concordant treatment were common within this community health system, as demonstrated by real-world data derived from the EMR. Data highlighted strengths in baseline molecular testing and treatment selection, while also identifying opportunities to improve documentation consistency and standardization of molecular testing workflows. Leveraging system-wide EMR to inform focus group discussions facilitated social learning and discussion, with the goal of further enhancing adherence to evidence-based AML management and improving guideline concordance across the health system, while recognizing that insights into workflows and treatment adoption are derived from a limited qualitative sample and should be interpreted with appropriate context.

## Appendices

Appendix A 

**Table 6:** Semi-structured focus group question guide FLT3: FMS-like tyrosine kinase 3, AML: acute myeloid leukemia, EMR: electronic medical record, TKD: tyrosine kinase domain

Core domain	Question
Workflows for ordering and documenting molecular testing	Walk us through your current workflow for ordering molecular testing at AML diagnosis. Who is typically responsible for ordering FLT3 testing, and at what point in the workup is it requested? Which assays or laboratories are typically used for FLT3 testing and related molecular workup, and what factors influence that choice?
How FLT3 results informed induction and relapse treatment decisions	How are FLT3 results communicated and documented in the EMR, and what challenges, if any, affect their visibility or accessibility during treatment planning? In what ways do FLT3 results influence frontline induction decisions in your practice?
Barriers to timely testing or guideline-concordant treatment	What barriers make it difficult to deliver guideline-concordant care for patients with FLT3-mutated AML in your setting? Are there points in the care pathway—such as referral, admission, transplant evaluation, or relapse—where molecular testing or treatment planning becomes less standardized?
Differences in decision-making across provider roles or sites	How are decisions made regarding the choice among FLT3 inhibitors (for example, midostaurin, gilteritinib, or quizartinib), and do these decisions vary by provider role, disease setting, or site? What process changes, EMR tools, educational resources, or consensus pathways would most improve FLT3 testing workflows and treatment consistency across the health system?
Perceptions of newer FLT3 inhibitors and areas needing additional guidance	What are your perspectives on using newer FLT3 inhibitors, particularly in lower-intensity regimens, post-transplant settings, or for TKD-only mutations?

Appendix B 

**Table 7:** Induction treatment patterns by year (N = 210) Clof: clofarabine, Cyt: cytarabine, Dec: decitabine, FLAG-IDA: fludarabine, cytarabine, idarubicin, granulocyte colony-stimulating factor, Gem: gemtuzumab ozogamicin, Gil: gilteritinib, HIDAC: high dose cytarabine, HMA: hypomethylating agent, LDAC: low dose cytarabine, Lipo Dau/Cyt: liposomal daunorubicin/cytarabine, MIDO: midostaurin, QUIZ: quizartinib, Ven: venetoclax

Year	2017	2018	2019	2020	2021	2022	2023	2024	Total
Treatment	N = 25 (%)	N = 25 (%)	N = 20 (%)	N = 24 (%)	N = 36 (%)	N = 30 (%)	N = 25 (%)	N = 25 (%)	N = 210 (%)
7+3	5 (20)	7 (28)	2 (10)	4 (16.7)	5 (13.9)	5 (16.7)	3 (12)	2 (8)	33 (15.7)
7+3+Gem	--	--	1 (5)	2 (8.3)	--	--	1 (4)	--	4 (1.9)
7+3+MIDO	4 (16)	2 (8)	--	--	1 (2.8)	1 (3.3)	4 (16)	2 (8)	14 (6.7)
7+3+QUIZ	1 (4)	--	--	--	--	--	--	1 (4)	2 (1)
7+3+Ven	--	--	--	--	--	1 (3.3)	--	5 (20)	6 (2.9)
7+3+Ven+Gem	--	--	--	--	--	--	1 (4)	1 (4)	2 (1)
7+3+Ven+Gil	--	--	--	--	--	--	--	1 (4)	1 (.5)
Clof/Cyt	--	--	--	--	1 (2.8)	1 (3.3)	--	--	2 (1)
Dec/Ven/Gem	--	--	--	--	1 (2.8)	1 (3.3)	--	--	2 (1)
FLAG-IDA	12 (48)	4 (16)	3 (15)	3 (12.5)	3 (8.3)	1 (3.3)	3 (12)	--	29 (13.8)
FLAG-IDA/Ven	--	--	1 (5)	--	1 (2.8)	--	2 (8)	--	4 (1.9)
FLAG-IDA/Ven/MIDO	--	--	--	--	--	1 (3.3)	--	--	1 (0.5)
HIDAC	--	--	--	--	--	1 (3.3)	--	--	1 (0.5)
HMA	1 (4)	6 (24)	4 (20)	1 (4.2)	2 (5.6)	--	--	--	14 (6.7)
HMA+MIDO	1 (4)	1 (4)	--	1 (4.2)	--	--	--	--	3 (1.4)
Lipo Dau/Cyt	1 (4)	4 (16)	6 (30)	4 (16.7)	4 (11.1)	2 (6.7)	3 (12)	--	24 (11.4)
Ven+HMA	--	1 (4)	3 (15)	7 (29.2)	17 (47.2)	15 (50)	7 (28)	13 (52)	63 (30)
Ven+HMA+Gil	--	--	--		--	1 (3.3)	1 (4)	--	2 (1)
Ven+HMA+MIDO	--	--	--	1 (4.2)	1 (2.8)	--	--	--	2 (1)
Ven+LDAC	--	--	--	1 (4.2)	--	--	--	--	1 (0.5)
